# Scene Graph and Natural Language-Based Semantic Image Retrieval Using Vision Sensor Data

**DOI:** 10.3390/s25113252

**Published:** 2025-05-22

**Authors:** Jaehoon Kim, Byoung Chul Ko

**Affiliations:** Department of Computer Engineering, Keimyung University, Daegu 42601, Republic of Korea; tyczend@gmail.com

**Keywords:** vision sensor, graph similarity learning, graph neural network, scene graph generation, semantic image retrieval, subgraph extraction

## Abstract

Text-based image retrieval is one of the most common approaches for searching images acquired from vision sensors such as cameras. However, this method suffers from limitations in retrieval accuracy, particularly when the query contains limited information or involves previously unseen sentences. These challenges arise because keyword-based matching fails to adequately capture contextual and semantic meanings. To address these limitations, we propose a novel approach that transforms sentences and images into semantic graphs and scene graphs, enabling a quantitative comparison between them. Specifically, we utilize a graph neural network (GNN) to learn features of nodes and edges and generate graph embeddings, enabling image retrieval through natural language queries without relying on additional image metadata. We introduce a contrastive GNN-based framework that matches semantic graphs with scene graphs to retrieve semantically similar images. In addition, we incorporate a hard negative mining strategy, allowing the model to effectively learn from more challenging negative samples. The experimental results on the Visual Genome dataset show that the proposed method achieves a top nDCG@50 score of 0.745, improving retrieval performance by approximately 7.7 percentage points compared to random sampling with full graphs. This confirms that the model effectively retrieves semantically relevant images by structurally interpreting complex scenes.

## 1. Introduction

Image retrieval using data acquired by vision sensors such as cameras is an active area of research in computer vision, with applications spanning search engines, medical image analysis, security and surveillance systems, and social media platforms. Traditional text-based image retrieval (TBIR) approaches utilize text annotations or tags associated with images to perform retrieval. However, these methods often fail to reflect the visual content of the images accurately and may result in the loss of important semantic information [1,2,3]. Pretrained large-scale vision–language models (VLMs) have recently been employed to generate textual descriptions from images for use in image retrieval. These generated captions are then used as inputs to retrieval models such as contrastive language–image pretraining (CLIP) [4] to perform image retrieval based on semantic similarity [5].

However, these methods face several challenges, including: (1) a strong dependence on captions generated by VLMs, (2) the risk of hallucinated or inaccurate captions, and (3) the lack of explicit modeling of inter-object relationships, all of which hinder accurate image retrieval.

To address the limitations of traditional TBIR approaches, content-based image retrieval (CBIR) has been proposed. However, CBIR methods also have limitations, as they rely solely on visual features and fail to capture object attributes and complex relationships among objects within scenes [6,7].

In this study, we propose a novel image retrieval system that transforms natural language queries into semantic graphs and evaluates their similarity to scene graphs constructed from an image database. While previous approaches have primarily relied on keyword matching or convolutional neural network (CNN)-based feature representations, our method leverages graph structures to more effectively capture inter-object relationships and contextual semantics, enabling a more precise similarity assessment.

**Figure 1:**
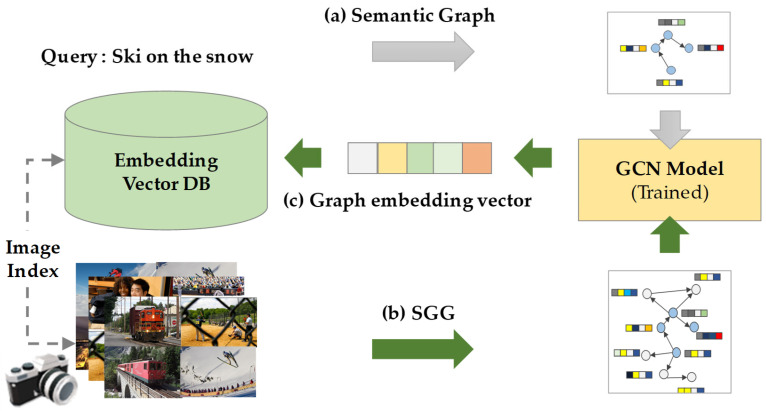
Overall architecture of the proposed semantic image retrieval system: (**a**) The input query sentence is first converted into a semantic graph; (**b**) scene graphs are generated from images acquired by various vision sensors through scene graph generation (SGG); (**c**) both the semantic graph and the scene graph are fed into a shared graph convolutional network (GCN), which encodes them into graph embedding vectors. These embeddings reside in a shared embedding space, allowing the system to quantitatively measure the similarity between text and image representations. The generated embedding vectors are stored in a database, enabling efficient retrieval.

As shown in Figure 1, the proposed framework retrieves semantically relevant images by generating a semantic graph from a natural language text query and matching it with a database of scene graphs extracted from images using a graph convolutional network (GCN). Scene graphs, in particular, serve as an effective tool for semantic representation, as they structurally encode complex image content into objects, attributes, and relationships. This structural abstraction enables more accurate similarity measurements compared to traditional keyword-based approaches.

For instance, a query such as “a person walking a dog” might only focus on the keywords “person” and “dog” in conventional keyword-based retrieval. In contrast, scene graph-based retrieval considers relational elements such as “walking” and “holding a leash”, leading to a more precise search outcome. Similarly, a query like “a person ordering food at a restaurant” may result in ambiguous matches when using simple keyword-based methods that emphasize only “person” and “food”. However, the use of scene graphs allows the system to capture relational semantics like “ordering food” and “interacting with a waiter”, thereby facilitating more semantically accurate retrieval.

### Contribution of This Work

In this paper, we propose a graph-based image retrieval system that transforms text queries and images into semantic graphs, enabling the retrieval process to incorporate inter-object relationships. This approach allows more precise contextual understanding compared to traditional keyword-based retrieval methods. The main contributions of this paper are as follows:

- A novel similarity measurement method for heterogeneous graph structures: We propose a technique that transforms scene graphs extracted from images and semantic graphs generated from text into embedding representations that preserve both node and edge information. By leveraging a GCN, our method enables quantitative similarity computation between the two graph structures.
- Subgraph extraction from region-based scene graphs: Scene graphs generated from images often contain a large number of objects and relationships, which leads to increased computational complexity and potential interference from irrelevant information during training. To address this, we propose a region-based subgraph extraction method that selectively retains only semantically meaningful relationships around a central node, thereby reducing graph processing overhead. By employing a window-based depth-first search (DFS) strategy, the method mitigates central node bias and captures localized semantic structures. These localized subgraphs are then merged to construct a semantically focused summary of the global scene graph. Experimental results demonstrate that the dataset generated using this method contributes to improved retrieval performance.
- An effective training strategy for text-to-image retrieval: We adopt a contrastive GCN-based framework that matches semantic graphs with scene graphs to retrieve semantically similar images. To further enhance learning, we incorporate hard negative mining (HNM) [8], allowing the model to focus on more challenging negative samples. Unlike conventional negative sampling methods that select samples randomly, HNM selects hard negatives—samples the model is more likely to confuse—after an initial training phase. This approach improves the model’s ability to distinguish subtle differences between similar images, leading to enhanced retrieval accuracy and better similarity evaluation performance relative to training time.

## 2. Related Work

### 2.1. Scene Graph Generation

A scene graph is a structured representation of visual information in the form of a graph, where nodes correspond to objects and their attributes, and edges represent relationships between objects. Scene graph generation (SGG) [9] refers to the process of analyzing an image to construct a scene graph, enabling the extraction of semantic data that captures complex inter-object relationships within the visual content.

### 2.2. Image Retrieval

Image retrieval has traditionally been categorized into two main approaches: TBIR and CBIR. TBIR relies on manually annotated textual metadata, such as tags or descriptions, to retrieve relevant images [1,2,3,4]. This method primarily depends on matching textual information rather than analyzing the visual characteristics of the image. However, it often fails to capture the actual visual semantics of the content, resulting in a potential loss of semantic information and limited retrieval performance.

CBIR retrieves images by leveraging the visual features extracted directly from the images themselves. Traditional CBIR methods rely on local features such as SIFT [10] and SURF [11] or utilize CNNs to extract visual representations for comparing query inputs with candidate images [12]. Although CBIR captures visual similarity effectively, it often struggles to represent complex semantic content, thereby limiting retrieval accuracy. To address this limitation, recent studies have proposed the use of deep learning-based VLMs to bridge the semantic gap between images and textual queries [5,13,14]. Notably, models like CLIP [4] learn joint representations of images and text, enabling retrieval based on semantic similarity between visual content and textual descriptions. These models substantially improve retrieval performance by aligning images with their contextual meanings. However, these approaches still face critical challenges. Because VLM-based retrieval often relies on generated captions or textual representations, inaccurate captioning can degrade retrieval performance. Furthermore, hallucination effects—where models produce plausible but incorrect captions—may compromise the reliability of retrieval outcomes. In addition, the current VLMs often fail to fully capture object-level interactions and contextual relationships, making it difficult to accurately align the semantics of an image with a textual query. Collectively, these limitations hinder the ability to perform precise and trustworthy image retrieval.

### 2.3. Subgraph Extraction

Scene graphs are designed to represent holistic relationships within an image; however, they often include excessive information, which increases computational complexity and complicates the identification of relevant content. In particular, scene graphs generated through SGG tend to exhibit fully connected structures, linking nearly all possible object pairs. In such cases, GCN-based models face challenges in selectively learning meaningful relationships, as the dense connections dilute semantic relevance. Consequently, existing subgraph extraction methods struggle to isolate the core semantic structures from these densely connected graphs.

To address this issue, we propose a relationship-aware subgraph extraction method that leverages a DFS strategy to traverse the scene graph. By selectively excluding semantically irrelevant nodes, our method constructs localized and balanced subgraphs that retain the essential relational structure while reducing noise and redundancy. This approach enables more effective and focused representation learning within graph-based models.

## 3. Preliminaries

### 3.1. Natural Language Processing

To convert textual queries into a graph structure, we utilize the natural language processing (NLP) toolkit spaCy [15] to perform syntactic parsing of input sentences. Based on the results of dependency parsing, we construct a directed graph where each word is represented as a node, and syntactic relationships between words are modeled as edges with directionality. Each node is further initialized using global vectors for word representation (GloVe) embeddings [16], providing a numerical representation of the word’s semantic meaning. This text graph construction serves as a preprocessing step to enable structural alignment with image scene graphs in subsequent stages of the framework.

### 3.2. Sentence-BERT (SBERT)

To quantitatively compare the semantic similarity between text and image graphs, we employ sentence-BERT (SBERT) [17] in our study. SBERT is a sentence embedding model built upon the bidirectional encoder representations from transformers architecture, specifically designed to generate fixed-length vector representations of sentences. These embeddings can be directly used for similarity computations such as cosine similarity or dot product.

In our framework, we utilize the sentence embeddings generated by SBERT to compute the semantic similarity between the semantic graph and the scene graph. This similarity score serves as a surrogate relevance signal [18], which is incorporated into the loss function during training. By integrating this measure, the GCN model is guided to learn more semantically aligned and meaningful representations.

### 3.3. SGG (Scene Graph Generation)

Semantic information can be effectively extracted from an image by representing object-level relationships in the form of a graph. To this end, we adopt the COOK model [19], which incorporates the concept of object co-occurrence into the graph structure. Scene graph generation enables the detection of objects, attributes, and relationships within an image, and represents them as a structured graph.

In our study, the scene graph is constructed as a directed graph, where all detected objects, their attributes, and inter-object relationships are represented as nodes. Each node is initialized with a 100-dimensional GloVe embedding, providing a dense vector representation of the corresponding word. This setup facilitates structured reasoning over the visual content and supports downstream tasks such as image–text alignment and semantic matching.

### 3.4. GCN (Graph Convolutional Network)

The GCN is a neural architecture designed to model relationships between nodes based on graph structures. Through convolution operations utilizing the adjacency matrix, GCNs iteratively update node representations by aggregating features from neighboring nodes. This process is grounded in spectral graph theory, enabling the network to effectively capture structurally connected semantic information. The learned node embeddings are typically aggregated using operations such as mean pooling to produce a fixed-size graph-level embedding, which can then be used to compute similarity scores via inner products or distance-based measures. The Siamese network [20], known for its effectiveness in measuring similarity between two inputs through shared weights, has also been widely adopted for comparing graph structures. Building on this concept, several graph neural network (GNN) architectures have been proposed to address the challenge of comparing graphs with different structures [21].

In this study, we propose using semantically focused subgraphs as input to the GCN, rather than processing the entire graph, to improve efficiency and relevance. By filtering out irrelevant information and concentrating on meaningful local structures, this approach enhances both the expressiveness of embeddings and the retrieval performance.

## 4. Semenatic Image Retrieal

We propose a novel framework that enables fine-grained semantic retrieval between textual queries and images by transforming both modalities into structured graph representations and comparing their structures using GCNs. While previous research [1,2,3,4,5,13,14] has predominantly focused on similarity retrieval between images, our approach extends semantic retrieval to align natural language queries with visual content. Instead of relying on global feature embeddings, as in traditional TBIR or CBIR, our method captures internal structures and relationships within queries and images through semantic graph representations. Although CLIP-based models [5,13,14] achieve strong performance through large-scale pretraining, our framework is designed to enable semantic retrieval without depending on CLIP, by focusing on direct structural matching. For evaluation purposes, however, CLIP is used as a reference point to compare retrieval performances.

**Figure 2:**
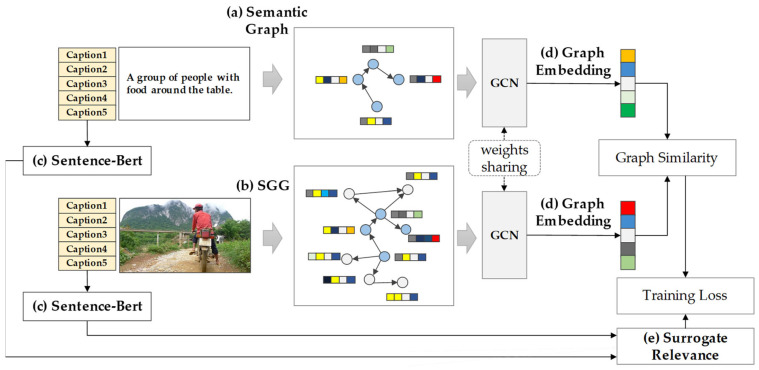
Training process using semantic scene graphs and contrastive GCN matching: (**a**) The search query is transformed into a semantic graph, (**b**) while the image is converted into a scene graph. (**c**) SBERT computes caption similarity without direct human labeling and quantifies the similarity between images. (**d**) The GCN model generates graph embedding and (**e**) uses the precomputed surrogate relevance for training.

Figure 2 illustrates the overall model training process, which leverages semantic scene graphs and contrastive GCN matching. As shown in Figure 2a, the retrieval query is text-based. The input sentence is processed using the natural language processing toolkit spaCy [15], which performs tokenization and extracts morphological, part-of-speech, and syntactic dependency information. For the image database, each image is automatically converted into a scene graph using a pretrained SGG model, as illustrated in Figure 2b. The SGG captures objects, their attributes, and inter-object relationships from the image, enabling the construction of structured semantic representations for image retrieval. Surrogate relevance [18], shown in Figure 2e, serves as an alternative metric to estimate semantic similarity without the need for manual human labeling. This is achieved using SBERT [17], which computes semantic similarity scores between image captions. These similarity scores are then used as alignment targets during the graph embedding learning process, guiding the model to produce semantically consistent retrieval results.

When multiple natural language queries describe the same visual content, surrogate relevance scores help identify semantically similar sentence pairs, which are used to inform the graph-level contrastive learning objective. This process enhances the model’s ability to learn fine-grained semantic similarity, ultimately improving retrieval performance.

### 4.1. Semantic Text Graph Construction

To convert a textual query into a graph structure, the input sentence is first tokenized into a sequence of words. Then, spaCy is used to analyze part-of-speech tags and dependency parsing information for each token, enabling the construction of a structured graph representation based on the syntactic relationships between words.

(1) $$S=\left\{\begin{array}{c} w_{1},w_{2},\ldots ,w_{n} \end{array}\right\}$$

To normalize variations in word forms caused by tense, number, and inflection, lemmatization is applied. Based on the lemmatized tokens, the node set is defined as follows:

(2) $$V=\left\{w_{i}^{\left(\mathrm{lemma}\right)}|w_{i}\in S\right\}$$

Based on the results of dependency parsing, directional edges are established between words to represent their syntactic relationships. The resulting semantic (text) graph is then formulated as follows:

(3) $$G=\left(V,E\right),\;E=\left\{\left(v_{i}\to v_{j}\right)|\mathrm{dependency}\left(v_{i},v_{j}\right)\right\}$$

Each node $v_{i}\in V$ is transformed into a *d*-dimensional vector representation $h_{i}\in {\mathbb{R}}^{d}$ using pretrained GloVe embeddings. The overall node feature matrix is then constructed as follows:

(4) $$H=\left[h_{1}^{T};\;h_{2}^{T};\;\ldots \;;\;h_{n}^{T}\right]\in {\mathbb{R}}^{n\times d},\;h_{i}=\mathrm{GloVe}\left(v_{i}\right)$$

where the matrix *H* serves as the input to the GCN and is utilized to learn the structural semantic representations of the text graph. Here, $v_{i}$ and $v_{j}$ represent nodes corresponding to lemmatized words. *n* denotes the number of words (nodes) in the input sentence. *d* denotes the dimensionality of the GloVe embeddings, which is set to 100 in this study.

### 4.2. Scene Graph Similarity

The structural similarity between the graph $G_{\mathrm{query}}$ generated from the text query, and the scene graph $G_{\mathrm{image}}$ constructed from the image, is defined in terms of the similarity between their semantic representations. This approach reformulates the problem of text–image semantic alignment as a similarity computation task based on graph embeddings, thereby enabling a structural comparison of heterogeneous input modalities within a unified graph representation space.

The similarity between graphs is computed via the dot product of their corresponding embedding vectors, as formulated below:

(5) $$Sim_{\mathrm{graph}}\left(G_{\mathrm{query}},G_{\mathrm{image}}\right)=z_{\mathrm{query}}^{⊤}z_{\mathrm{image}}$$

where $z_{\mathrm{query}}$ and $z_{\mathrm{image}}$ are fixed-dimensional vectors obtained by applying mean pooling to the embeddings of the query and image graphs, respectively, after processing them through a GCN. Each graph embedding vector can be defined as follows:

(6) $$z_{\mathrm{query}}=\mathrm{MeanPooling}\left(\mathrm{GCN}\left(G_{\mathrm{query}}\right)\right),\;z_{\mathrm{image}}=\mathrm{MeanPooling}\left(\mathrm{GCN}\left(G_{\mathrm{image}}\right)\right)$$

The embeddings of image scene graphs can be precomputed and stored in advance, allowing efficient similarity comparison by computing the query graph embedding in real time when a query is given.

### 4.3. Pair Sampling and Training Loss

A major practical limitation in learning semantic alignment between images is that the number of image pairs required for training grows quadratically as $O\left(N^{2}\right)$ with the size of the dataset. This issue becomes more prominent in real-world retrieval systems, where new images are continuously added, making repeated training over the entire dataset highly inefficient in terms of scalability. This challenge is analogous to the indexing structure in information retrieval systems, highlighting the need for a selective sampling strategy that constructs semantically meaningful training pairs while maintaining or improving retrieval performance. In this study, we address this requirement by designing a training pair sampling strategy based on surrogate relevance between images and define a corresponding training loss function.

(1) Definition of Surrogate Relevance

Each image $I_{i}$ and $I_{j}$ is associated with *n* captions, denoted as $c_{i}^{\left(k\right)}$ and $c_{j}^{\left(l\right)}$, respectively. For each caption, the embeddings $h_{i}^{\left(k\right)}$ and $h_{j}^{\left(l\right)}$ are obtained using SBERT [17]. The surrogate relevance $r_{ij}$ between the two images is then defined as the average cosine similarity between all pairs of caption embeddings, as formulated below:

(7) $$r_{ij}=\frac{1}{n^{2}}\sum_{k=1}^{n}\sum_{l=1}^{n}\cos\left(h_{i}^{\left(k\right)},h_{j}^{\left(l\right)}\right)$$

This serves as a proxy measure that reflects actual text-based semantic similarity and is utilized as a training label.

(2) Uniform Pair Sampling (Initial Training Strategy)

In this study, uniform pair sampling (UPS) refers to a strategy in which image pairs are classified as positive or negative based on their surrogate relevance $r_{ij}$, and training pairs are then constructed by randomly sampling from each group at an equal 1:1 ratio. This approach helps maintain a balanced distribution between positive and negative pairs during the early stages of training, enabling the model to learn stable and discriminative representations.

(3) Hard Negative Mining (Progressive Strategy)

Once the initial representation learning has reached a certain level of convergence, an HNM strategy is employed to facilitate more fine-grained representation learning. HNM prioritizes the selection of negative pairs with low surrogate relevance but high graph embedding similarity, thereby encouraging the model to better distinguish subtle semantic differences during training:

(8) $$r_{ij}<\tau \;\mathrm{and}\;{\mathrm{Sim}}_{\mathrm{graph}}\left(G_{i},G_{j}\right)>\delta$$

where *τ* denotes the upper bound of surrogate relevance, and δ  represents the lower bound of graph similarity. Hard negative pairs are selected as a subset of all negative pairs, with their sampling ratio gradually increasing as training progresses. This progressive hard negative sampling strategy aligns with the principles of curriculum learning, offering a balance between training stability and effectiveness.

(4) Definition of Loss Function

The model is trained to ensure that the predicted graph embedding similarity ${\hat{r}}_{ij}$ approximates the ground truth surrogate relevance $r_{ij}$. The loss function is defined using mean squared error (MSE), as follows:

(9) $$L=\frac{1}{|B|}\sum_{\left(i,j\right)\in B}{\left({\hat{r}}_{ij}-r_{ij}\right)}^{2}$$

where B denotes the set of training pairs within a batch. This training strategy enables efficient representation learning without the need for repeated training on the entire dataset. As a result, it facilitates the construction of an optimized framework that reduces training time while maintaining retrieval performance.

## 5. Proposed Subgraph Extraction

### 5.1. Motivation for Subgraph-Based Representation

Scene graphs generated from images, which include both objects and their relations, provide rich visual semantics. However, utilizing the entire graph during training is computationally expensive and may introduce semantically irrelevant information, potentially hindering the model’s generalization performance. In particular, the number of detected objects in an image can vary significantly, and there is often an imbalance in the spatial distribution and sizes of these objects. Such data disparities are reflected in the structure of the scene graph, introducing noise during training.

In this paper, we propose a subgraph extraction method called region-based semantic subgraph extraction (R-SSE). This method involves selecting region-based central nodes and performing a constrained DFS to extract minimal subgraphs, which are then merged. The approach is inspired by the scene graph decomposition method proposed by Zhong et al. [22], which highlights that scene graphs can vary significantly in structure depending on object count and relation density and that excessive connectivity may hinder learning.

R-SSE mitigates this issue by selecting semantically meaningful nodes within local regions of the image and constructing local minimal subgraphs that include only the most relevant relations. This strategy enhances both training efficiency and generalization performance by focusing learning on essential visual semantics.

**Figure 3:**
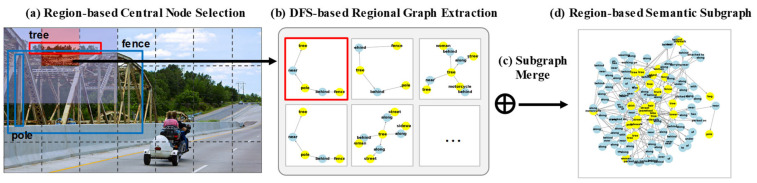
Proposed subgraph extraction method (R-SSE): (**a**) The input image is divided using a sliding window, and a central node is selected within each window; (**b**) directed depth-first search (DFS) is performed to extract local subgraphs; (**c**) overlapping nodes are merged to integrate the subgraphs; (**d**) Finally, region-based semantic subgraphs are constructed.

The overall processing pipeline of the proposed R-SSE is illustrated in Figure 3. It visually demonstrates the process of selecting central nodes from the input image, constructing local subgraphs via a directed DFS, and merging them to form the final semantically focused subgraph.

### 5.2. R-SSE (Region-Based Semantic Subgraph Extraction)

(1) Region-based Central Node Selection

To perform a regional partitioning of the nodes in a scene graph $G=\left(V,E\right)$, a sliding window technique is applied. A window region W of fixed size is moved across the image in a grid-like manner, and the set of nodes within each window is denoted as $V\left(W\right)$. Within each window, the central node c is selected as the object with the largest spatial extent, measured by its area.

This selection process is formally defined as:

(10) $$c=\arg\underset{v\in V\left(W\right)}{\max}\mathrm{Area}\left(v\right)$$

where $\mathrm{Area}\left(v\right)$ denotes the bounding box area representing the spatial extent of object v. Selecting the object with the largest area aims to prioritize visually prominent objects, which are likely to carry higher semantic significance within the scene. In addition, a set of neighborhood windows $N\left(W\right)$ is defined around the central window W. Subsequent exploration is restricted to the nodes located within $N\left(W\right)$, excluding those in the central window itself. This design reduces the bias toward the central node and enables the selection of semantically relevant external nodes, thereby enhancing the representational quality of the extracted subgraph.

(2) DFS-Based Regional Graph Extraction

A local subgraph $G_{c}=\left(V_{c},E_{c}\right)$ is constructed with the central node c as its starting point. The exploration is performed on the directed scene graph G using a constrained DFS strategy. The search constraints are defined as follows:

- Maximum depth: d=2;
- Maximum number of nodes: m=5 (including the central node);
- Search node restriction: $V_{\mathrm{search}}=\left\{u\in V\left(N\left(W\right)\right)\right\}$.

This exploration method incorporates both semantic relationships and spatial constraints, rather than relying solely on location-based expansion. As a result, it enables the extraction of local yet semantically meaningful subgraphs.

The overall procedure is detailed in Algorithm 1 of this paper, which includes the selection of region-based central nodes via a sliding window, exploration of surrounding window nodes, DFS-based expansion, and condition-based termination. The maximum number of nodes m is set to 5, including the central node, in order to maintain the compactness of the local subgraphs and to focus on capturing the most semantically relevant objects within each region. The sliding window size is empirically set to 75 pixels to balance local semantic concentration and spatial coverage. The maximum exploration depth d is set to 2 to maintain semantic locality and avoid overly deep traversals.

(3) Subgraph Merge

In the previously defined sliding window-based subgraph generation process, a local minimal subgraph is extracted from each region, centered around a central node and composed of semantically connected objects. These minimal subgraphs independently represent localized relationships across different spatial regions of the image and enable the model to learn distinct semantic units that may otherwise be entangled within a global scene graph.

This section describes the procedure for merging multiple local subgraphs—generated during the execution of Algorithm 1—into a single, unified subgraph. The core idea of the merging process is to normalize and consolidate duplicate object nodes that may appear across different local subgraphs. Specifically, nodes referring to the same object in different subgraphs are merged into a single unified node. The edges between these merged nodes preserve the original structural relations from their respective subgraphs, thereby forming a new, integrated semantic subgraph.

- *V*_merge: the normalized set of nodes obtained by removing duplicates across all merged nodes;
- *E*_merge: the set of edges preserving the original relationships among the merged nodes.

(11) $$G_{\mathrm{merge}}=\left(V_{\mathrm{merge}},E_{\mathrm{merge}}\right)$$

Through this merging process, a focused representation composed only of semantically relevant objects can be extracted from the overall scene graph. This enables the construction of subgraphs that are locally dense in information while eliminating unnecessary or excessively connected components from the complex global graph structure. By reducing the over-complexity of the full scene graph and providing a semantically condensed representation, this structure introduces an effective inductive bias for GNN-based learning.
**Algorithm 1.** Region-based Semantic Subgraph Extraction with Subgraph Merge (R-SSE)**Input:** Scene graph $G=\left(V,E\right)$, sliding windows W, max node count m,   max depth d
**Output:** Merged subgraph $G_{\mathrm{merge}}=\left(V_{\mathrm{merge}},E_{\mathrm{merge}}\right)$
1: S←Ø  // Set of all regional subgraphs 2: for each center window W∈W do 3: c← node in $V\left(W\right)$ with the largest blob area 4: $V_{c}\leftarrow \left\{c\right\}$
5: Stack $\leftarrow \left[\left(c,0\right)\right]$ 6: while Stack is not empty and $\left|V_{c}\right|<m\;do$ 7: $\left(v,\mathrm{depth}\right)\leftarrow$ Stack:pop() 8: for each $\left(v\to u\right)\in E$ do 9: if $u\in V\left(N\left(W\right)\right)$ and $u\notin V_{c}$ and depth+1≤d and 10: $\left|V_{c}\right|<m$ then 11.$\;\;\;\;\;\;\;\;\;\;V_{c}\leftarrow V_{c}\cup \left\{u\right\}$ 12: Stack:push(u,depth+1) 13: $E_{c}\leftarrow \left\{\left(u\to v\right)\in E|u,v\in V_{c}\right\}$ 14: $G_{c}\leftarrow \left(V_{c},E_{c}\right)$ 15: $S\leftarrow S\cup \left\{G_{c}\right\}$ 16: $V_{\mathrm{merge}}\leftarrow$ union of all nodes in S, duplicates removed 17: $E_{\mathrm{merge}}\leftarrow$ all edges among $V_{\mathrm{merge}}$ from E 18: **Return** $G_{\mathrm{merge}}=\left(V_{\mathrm{merge}},E_{\mathrm{merge}}\right)$


## 6. Dataset and Experimental Results

### 6.1. Dataset and Sampling Strategy

In this study, we constructed the experimental dataset based on the publicly available aaai2021-scene-graph-image-retrieval dataset [23], which was originally built by selecting semantically similar images and captions from the MS-COCO [22] dataset. Semantic similarity in the original dataset was determined through a combination of human annotations and SBERT-based semantic similarity scores between captions. A total of 3216 images were included in the dataset.

To enable graph-based structural semantic retrieval research, we further extended the dataset by incorporating scene graph annotations from the Visual Genome [24] dataset for each corresponding image. Each training sample is organized as a pair consisting of a scene graph and a caption graph derived from the same image. For example, if image *A* has the caption “A cat sits on a mat”, the sentence is converted into a graph structure and paired with the corresponding scene graph extracted from the image for training purposes.

The total number of images used in the experiment is 3216, and with five captions per image across all samples to maintain consistency and fairness in training, as provided by the MS-COCO dataset. This yields a total of 3216 × 5 × 3216 training pairs. To ensure efficient training, only 25% of these pairs were used during model training. For evaluation, the first caption associated with each image (3216 captions in total) was used as query data to assess retrieval performance.

Semantic similarity between text and image modalities was learned based on these training pairs. Because the quality of pair construction significantly impacts final performance in contrastive learning frameworks, additional experiments were conducted to analyze performance differences according to the pair sampling strategies. The training pairs were constructed using three different sampling methods.

The dataset for comparative experiments was constructed by utilizing caption information from MS-COCO and scene graph annotations from Visual Genome. Because the proposed method generates semantic graphs from captions and extracts scene graphs from images, it can be applied to any dataset that provides both images and caption information. Therefore, the framework is designed to be applicable to other datasets that satisfy these conditions.

(1) Random Sampling:

Image pairs are randomly selected without considering similarity and are then used to construct positive and negative pairs. While this approach is straightforward, it lacks control over training difficulty and may result in a high proportion of easy negative samples, potentially reducing training efficiency.

(2) Uniform Pair Sampling (UPS):

Image pairs are classified as positive or negative based on their surrogate relevance scores, and training pairs are then constructed by randomly sampling from each category at a 1:1 ratio. This method is employed during the early stages of training to ensure stability in representation learning while maintaining data diversity.

(3) Hard Negative Mining (HNM):

Negative pairs are selected such that they exhibit low surrogate relevance but high graph structural similarity. By including these hard negative samples—those that are challenging for the model to distinguish—this strategy enhances the precision of representation learning and contributes to improved generalization performance. HNM is not applied during the initial stages of training but is introduced at a fixed ratio after a certain number of training epochs.

### 6.2. Training Details

The Adam optimizer is employed with an initial learning rate of 0.0001 and a weight decay of 5 × 10^−4^. The batch size is set to 384, and the model is trained for a total of 20 epochs.

### 6.3. Evaluation

In this study, normalized discounted cumulative gain (nDCG) is employed to quantitatively evaluate the performance of text-to-image retrieval. nDCG is a representative ranking-based evaluation metric that assigns higher weights to items appearing at the top of the retrieved list, effectively capturing the balance between relevance and ranking position. The metric is defined as follows:

Discounted Cumulative Gain (DCG):

(12) $$DCG_{k}=\sum_{i=1}^{k}\frac{rel_{i}}{\log_{2}\left(i+1\right)}$$

Normalized NCG (nDCG):

(13) $$nDCG_{k}=\frac{DCG_{k}}{IDCG_{k}}\;\;$$

where $rel_{i}$ denotes the surrogate relevance score of the image retrieved at rank *i*, and $IDCG_{k}$ represents the ideal DCG value for the given query. In this experiment, nDCG@k for k = 10, 20, 30, 40, 50 is used as the primary evaluation metric. nDCG effectively reflects the alignment between the ranking of retrieved images and their surrogate relevance scores, making it a practical and reliable measure of retrieval system performance.

(1) Baseline Methods

The performance of the proposed model was compared against the following three baseline methods:

- Keyword Match: A simple method that computes similarity based on the number of matching words between the text query and image captions. This approach does not account for structural or semantic relationships between words and therefore yielded the lowest performance.
- Contrastive Language–Image Pretraining (CLIP): Similar to the method proposed by Karthik et al. [5], this baseline utilizes a pretrained model based on ResNet and Vision Transformer (ViT) to directly learn semantic similarity between text and images. Among the text-based methods, CLIP achieved the highest performance in this experiment.
- Ground Truth Scene Graph (GT/SG): This approach uses manually annotated scene graphs provided by Visual Genome without modification. By leveraging accurate object and relation information, this method achieved the best performance among the graph-based approaches.

(2) Scene Graphs

Table 1 presents a quantitative comparison of text-to-image retrieval performance across different graph representation methods and GNN architectures. Experimental results show that the GCN (Node) approach consistently outperforms others across all evaluation points (nDCG@10 to @50), achieving a score of 0.738 at nDCG@50. This represents an improvement of approximately 7.2 percentage points over graph attention networks (GATs) [25] (Node) and 0.4 percentage points over the GCN (Edge) under the same graph representation scheme.

The observed performance gap can be attributed to the structural differences between GATs and GCNs. While GATs learn the importance of neighboring nodes through an attention mechanism, GCNs aggregate information from neighboring nodes using a normalized, fixed weighting scheme. In this experiment, the normalized aggregation of GCNs proved more robust in complex or noisy graph environments than the flexibledk weighting of GATs. This suggests that in scene graphs, where relational density is often imbalanced or certain nodes are overly connected, attention weights in GATs may become skewed, leading to biased representation learning.

Furthermore, performance differences were observed across graph representation strategies. The edge-based approach (GCN Edge) represents relations solely as edges, which limits the extent to which relational information is incorporated into node embeddings. In contrast, the node-based approach (GCN Node) explicitly models relationships as separate nodes, enabling the GCN to learn embeddings for both objects and relations. This led to better performance; for instance, in the GT-based GCN setting, the node-based method achieved 0.738 at nDCG@50, while the edge-based method reached 0.734, with the node-based model consistently outperforming across all evaluated ranks (k = 10 to 50).

On the other hand, performance dropped when using automatically generated scene graphs (I/SG) compared to manually annotated ground truth scene graphs (GT/SG). For example, the GCN (Node) model achieved 0.738 on GT/SG, whereas it scored 0.668 on I/SG—a decrease of approximately 7.0 percentage points. This performance drop—from 0.738 (GT/SG) to 0.668 (I/SG) in GCN (Node) models—reflects the limitations of automatically generated scene graphs, which tend to include over-detected objects and redundant relationships. These artifacts lead to overly dense graph structures that reduce semantic diversity and introduce noise, thereby impairing the generalization ability of the GCN. To address this, we propose a combination of improved pair sampling and subgraph-based representation learning (Table 2) in Section 6.3, which demonstrably mitigates these issues and recovers performance close to that of GT/SG.

(3) Sampling Pair

The sampling strategy for training data plays a critical role in the performance of contrastive learning-based models. Through prior experiments, the limitations of random sampling were identified, and it was observed that the importance of negative pairs increases as training progresses. Accordingly, various sampling strategies were compared, and the results are summarized in Table 2. First, UPS involves separating all samples into positive and negative categories based on their surrogate relevance scores and then sampling an equal number of pairs (1:1 ratio) from each category. This method is particularly effective in the early stages of training, as it ensures representation stability and data diversity. Based on the full graph setting, UPS achieved an nDCG@50 of 0.714, showing an improvement of approximately 4.6 percentage points over random sampling (0.668).

In addition, the combination strategy UPS + HNM augments UPS with HNM, which progressively increases training difficulty by selecting negative pairs that are semantically dissimilar but have similar surrogate relevance scores. Under the full graph setting, UPS + HNM slightly outperformed UPS, achieving an nDCG@50 of 0.717, representing a 0.3 percentage point improvement. More notably, the performance gap was more pronounced in the subgraph-based setting. In particular, when UPS + HNM was applied in conjunction with the R-SSE subgraph extraction method, the model achieved the highest performance across all experimental conditions, recording an nDCG@50 of 0.745.

The Top-k approach constructs subgraphs by selecting the Top-k relationships based on the similarity ranking of node pairs predicted by the SGG module. While this method demonstrated improved performance compared to the full graph, a performance drop was observed as the number of selected nodes increased. For example, Top-k (k = 40) achieved a high nDCG@50 score of 0.739, but performance declined to 0.735 at Top-k (k = 80). This suggests that excessive increases in the number of nodes and edges may raise graph complexity, leading to potential overfitting or degradation in generalization during training.

In contrast, the proposed R-SSE method consistently outperformed the Top-k approach across all evaluation points (nDCG@10 to @50). R-SSE offers structural advantages by effectively capturing region-based semantic representations while maintaining a controlled number of nodes. On the other hand, the Top-k method demonstrates optimal performance only at specific values of k (e.g., k = 40), indicating limitations in its general applicability.

It is also worth noting that the evaluation benchmark used in this study—MS-COCO—provides natural language captions that typically describe the image as a whole. As a result, the advantages of R-SSE, which focuses on localized semantic representation, may not be fully captured in this setting. Nevertheless, R-SSE possesses structural properties that allow it to effectively capture semantic information from local regions within an image. Furthermore, it offers flexibility in adjusting semantic granularity through configurations such as the exploration scope and central node selection. These characteristics are expected to yield greater benefits in future application scenarios such as region-level scene queries or fine-grained scene descriptions.

(4) Subgraph Structure Analysis

**Figure 4:**
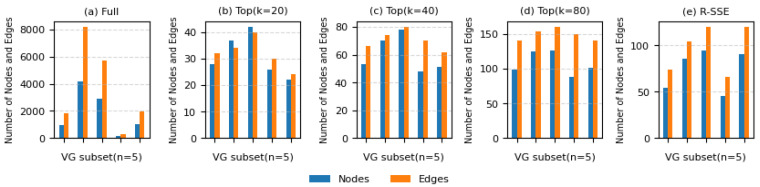
Comparison of node and edge counts for different subgraph extraction methods over five Visual Genome subset scenes: (**a**) Full, (**b**) Top (k = 20), (**c**) Top (k = 40), (**d**) Top (k = 80), (**e**) R-SSE.

To analyze the structural complexity introduced by different subgraph generation methods, we compared the number of nodes and edges in subgraphs generated from the same image set (a subset of Visual Genome, *n* = 5) using the ‘Full’, ‘Top-k’ (with k = 20, 40, 80), and the proposed R-SSE methods. Figure 4 visualizes the structural sizes of the subgraphs produced by each method, presenting the distribution of average node and edge counts.

The ‘Full’ method includes all possible relations between detected objects, resulting in an excessively dense structure with significantly increased graph size and computational overhead. In contrast, (*b*–*d*) the ‘Top-k’ methods limit graph size by selecting only the Top-k ranked relations; however, they exhibit large variations in the number of nodes and edges across images, indicating a lack of structural consistency. Notably, as k increases, the number of nodes tends to saturate, while the number of edges continues to grow proportionally. This suggests the inclusion of an excessive number of redundant or unnecessary connections.

The proposed R-SSE method constructs a more balanced graph by selecting region-based central nodes and semantically related objects within a constrained scope, effectively suppressing excessive connectivity while preserving only the most essential structure. Experimental results show that R-SSE consistently maintains stable levels of both node and edge counts, striking a balance between graph compactness and representational richness. These structural characteristics indicate that R-SSE provides an efficient subgraph structure that supports both computational efficiency and expressive power during model training.

While R-SSE introduces sliding windows and DFS traversal, these operations are confined to local subgraph segments with significantly reduced node counts (83.0 nodes on average compared to 1140.9 in full scene graphs). Moreover, sliding windows are applied only to Top-k object regions, avoiding exhaustive search. Although these steps are performed during offline preprocessing, they contribute to generating compact and semantically focused subgraphs. As shown in Table 3, this structure results in efficient training performance, with R-SSE requiring 83 ms per iteration—an approximately 16% increase compared to Top (k = 40)—while making it effective for controlled subgraph construction depending on task objectives.

### 6.4. Qualitative Results

**Figure 5:**
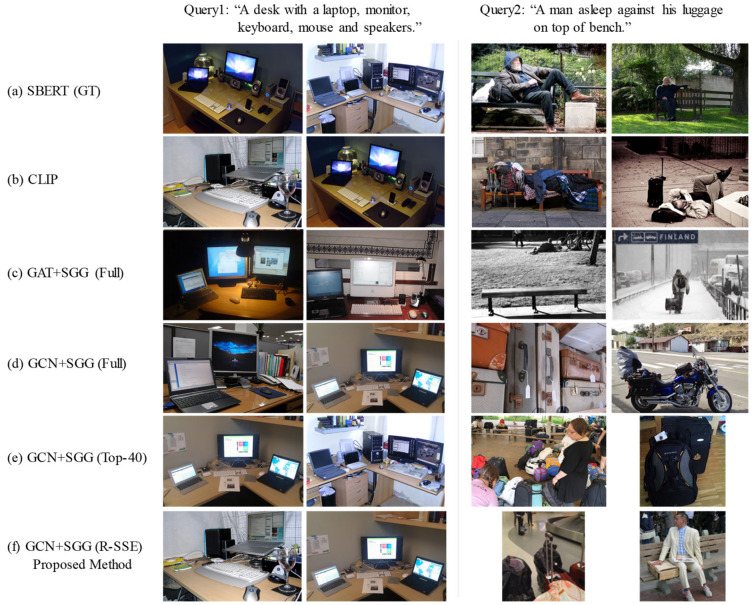
Comparison of retrieval results and methods: high similarity results (Query 1) vs. low similarity results (Query 2). (**a**) SBERT (GT), (**b**) CLIP, (**c**) GAT + Full, (**d**) GCN + Full, (**e**) GCN + Top-40, and (**f**) GCN + R-SSE (proposed).

To qualitatively evaluate the semantic alignment performance of the proposed model, we conducted a visual analysis comparing the top retrieved results for two natural language queries. Figure 5 presents the retrieval outcomes using different methods: (a) SBERT (GT), (b) CLIP, (c) GAT + Full, (d) GCN + Full, (e) GCN + Top-40, and (f) GCN + R-SSE

For Query 1 (“A desk with a laptop, monitor, keyboard, mouse and speakers.”), most models successfully retrieved semantically relevant desk images. However, for Query 2 (“A man asleep against his baggage on top of bench.”), CLIP retrieved relatively well-matched images, while the GCN + SGG model returned less relevant results. While overall performance remained limited, the proposed R-SSE (f) was the only graph-based method to retrieve images containing key objects—man, bench, and baggage—indicating its relative advantage in handling complex semantic queries. This discrepancy can be attributed to two main factors.

First, CLIP, having been pretrained on large-scale image–text pairs, demonstrates strong generalization across diverse expressions. In contrast, the performance of the GCN + SGG model is heavily dependent on the quality of the graph structures generated via SGG and the training pairs.

Second, the current SGG system constructs scene graphs based only on a predefined set of object and relation categories, limiting its ability to fully capture the semantic richness of the input query. This can result in misalignment between the semantic structure of the query and that of the scene graph. Furthermore, as contrastive learning relies on diverse and informative training samples, insufficient variation in representation may lead to degraded generalization performance.

To overcome these limitations, future work should consider incorporating visual features into the scene graphs generated by SGG through a multimodal graph representation approach. By integrating CNN- or ViT-based visual information into node embeddings, the expressive power of graph-based semantic alignment models can be significantly enhanced, thereby improving the model’s ability to learn semantic similarity between text and images.

## 7. Conclusions

In this paper, we proposed a method to enhance text-based image retrieval by learning semantic structural similarity between scene graphs and natural language queries. Existing image–text alignment methods often rely on global representations of entire images or, when using graph structures, fail to fully capture their underlying semantics. We designed a GCN-based model to address these limitations by learning the structural similarity between scene graphs generated from images and semantic graphs converted from text. We further introduced an R-SSE using a sliding window approach, as well as an HNM-based sampling strategy.

Through quantitative experiments, we demonstrated that both the graph representation method and the sampling strategy significantly influence model performance. The proposed method achieved a top nDCG@50 score of 0.745, improving retrieval performance by approximately 7.7 percentage points compared to random sampling with full graphs. In addition, qualitative analysis revealed structural limitations of the current approach and outlined directions for future improvement. In particular, we identified the limited expressiveness of automatically generated scene graphs and the lack of diversity in training samples as key factors contributing to performance degradation.

While our model demonstrates competitive performance across many natural language queries, we observe that it performs sub-optimally on complex or abstract queries such as “A man glides along a frozen lake”, as illustrated in Figure 5. This is primarily due to the limited scale and diversity of our training dataset (3216 images), which contrasts with the large-scale web-scraped pretraining data used in CLIP. Nevertheless, our model achieves strong performance on a wide range of grounded and compositionally simpler queries, highlighting its effectiveness in data-constrained settings.

We acknowledge that reliance on predefined relationship categories may limit the capacity to capture free-form or abstract semantics. To mitigate this, we integrate relation augmentation using CLIP-based similarity and employ soft textual–visual alignment during matching. Future work will explore structure-free or latent graph models to enable finer semantic matching.

## Figures and Tables

**Table 1:** Semantic image retrieval performance comparison across various GNN architectures and graph representation types using random sampling. “Caption” refers to ground truth textual annotations from the MS-COCO dataset, used as input for text-based models such as CLIP and Keyword Match. “GT/SG” denotes ground truth scene graphs from Visual Genome, and “I/SG” indicates automatically generated scene graphs via SGG.

Methods	Data	nDCG@ 10	nDCG@ 20	nDCG@ 30	nDCG@ 40	nDCG@ 50
Keyword Match	Caption	0.205	0.213	0.218	0.223	0.229
CLIP	Caption	0.845	0.846	0.845	0.844	0.844
GAT (Node)	GT/SG	0.628	0.644	0.653	0.660	0.666
GAT (Edge)	GT/SG	0.632	0.648	0.657	0.664	0.669
GCN (Node)	GT/SG	0.681	0.705	0.718	0.729	0.738
GCN (Edge)	GT/SG	0.676	0.700	0.715	0.725	0.734
GCN (Node)	I/SG	0.625	0.641	0.649	0.658	0.668
GCN (Edge)	I/SG	0.624	0.638	0.649	0.658	0.662

**Table 2:** Comparison of semantic image retrieval performance across subgraph extraction methods and sampling strategies. ‘Full’ uses the entire scene graph, while ‘Top(k)’ selects the Top-k relations based on predicted similarity. R-SSE is the proposed method focusing on localized semantic subgraphs. The best performance is achieved by combining R-SSE with UPS + HNM, highlighting the effectiveness of both structured subgraph selection and hard negative sampling.

Subgraph Extraction	Sampling Pair	nDCG@ 10	nDCG@ 20	nDCG@ 30	nDCG@ 40	nDCG@ 50
Full	Random	0.625	0.641	0.649	0.658	0.668
Full	UPS	0.680	0.695	0.704	0.710	0.714
Top (k = 20)	UPS	0.696	0.711	0.721	0.727	0.732
Top (k = 40)	UPS	0.697	0.716	0.726	0.733	0.739
Top (k = 80)	UPS	0.691	0.710	0.721	0.729	0.735
R-SSE	UPS	0.697	0.715	0.725	0.733	0.740
Full	UPS + HNM	0.679	0.697	0.707	0.713	0.717
Top (k = 40)	UPS + HNM	0.699	0.718	0.728	0.735	0.740
**R-SSE**	**UPS + HNM**	**0.704**	**0.722**	**0.732**	**0.740**	**0.745**

**Table 3:** Summary of the average preprocessing and training times for each subgraph extraction method.

Step	Full	Top (k = 20)	Top (k = 40)	Top (k = 80)	R-SSE
Preprocessing (Subgraph)	4 ms	0.1 ms	0.24 ms	0.5 ms	8 ms
Training (Iteration)	917 ms	54 ms	71 ms	125 ms	83 ms

## Data Availability

Data are contained within the article.
